# Problematic Media Use among Children up to the Age of 10: A Systematic Literature Review

**DOI:** 10.3390/ijerph20105854

**Published:** 2023-05-17

**Authors:** Valeria Rega, Francesca Gioia, Valentina Boursier

**Affiliations:** Department of Humanities, University of Naples Federico II, 80100 Naples, Italy; valeria.rega@unina.it (V.R.); valentina.boursier@unina.it (V.B.)

**Keywords:** problematic media use, children, systematic review

## Abstract

Introduction: Digital screen media use has significantly grown in all age groups and at an increasingly young age, including toddlers, schoolers, and primary school children. Although there is evidence that excessive early childhood media exposure can lead to several negative developmental outcomes, no systematic review on Problematic Media Use (PMU) of children under 10 years old have been provided. The aim of the present systematic review was to identify (i) the main instruments used to measure children’s PMU across different studies; (ii) the risk and protective factors which might increase or reduce children’s PMU; and (iii) the negative outcomes associated with children’s PMU. Methods: This study was conducted following the systematic review guidelines proposed in the PRISMA statement. A total of 35 studies published between 2012–2022 and with a mean sample age between 0 and 10 years old were ultimately included in this literature review. Results: Use of media for more than 2 h a day, male gender, and higher age increased the risk of developing PMU among children. PMU led to several negative consequences for children’s development and well-being (e.g., more problematic behaviors, sleep problems, higher depressive symptoms, lower emotional intelligence, and lower academic achievements). Children who experienced negative psychological symptoms, a dysfunctional parent–child relationship, and difficulties in school context were more prone to develop PMU. However, an authoritative parenting style and restrictive parental mediation reduced the risk of developing PMU among children. Finally, self-report measures specifically designed to get the younger children’s perspective are still few and not so widely used. Conclusions: Overall, this research field is still in its infancy and needs further investigation. Likely, a dysfunctional family system can lead children to experience emotional distress and negative psychological symptoms, which they try to manage by escaping into the virtual world, thus increasing the risk of developing PMU. As the children’s PMU is closely affected by the family environment, future prevention interventions should target both children and their parents to improve their self-regulatory and mentalizing capabilities, as well as parental mediation strategies and general parenting practices.

## 1. Introduction

In the last ten years, the percentage of children and adolescents aged 9 to 16 using digital screens (especially smartphones) to access the Internet has exceeded 80% in around 11 European countries, and daily time spent online has doubled from 1 to more than 2 h [1,2]. The use of digital media devices, especially smartphones and tablets, has also grown among toddlers, preschoolers, and primary school children aged 0 to 8 [3], with 15% of children having access to their own mobile phone from the age of 3 [4]. Recently, repeated lockdowns due to the COVID-19 pandemic and the related distance learning has inevitably affected young individuals’ digital habits, leading to more time spent in indoor and online activities than before, with serious effects on physical and psychological health [5,6]. Consequently, from 2020 to date, the number of studies aimed at evaluating the prevalence of excessive, problematic, or addictive Internet use across different age groups, as well as the related negative outcomes, has grown exponentially [7,8].

Over the last two decades, several labels have been used to describe Internet overuse, such as Internet addiction [9,10,11], problematic Internet use [12], and pathological Internet use [13], thus reflecting the lack of scientific consensus on how this phenomenon should be theoretically defined, operationalized, and measured [14,15]. Indeed, only Internet Gaming Disorder has been included in Section III of the 5th edition of Diagnostic and Statistical Manual of Mental Disorder (DSM-5) among the conditions requiring further investigation and clinical-empirical research [16]. More recently, the World Health Organization [17] introduced Gaming Disorder in the 11th Revision of the International Classification of Diseases (ICD-11). On the contrary, Internet Addiction Disorder (IAD) has not yet been included in any diagnostic manual of mental disorders.

Although media device use has become increasingly widespread across all age groups and even among younger children, most theoretical and empirical studies on problematic online behaviors, such as Internet addiction [18,19,20,21], problematic Internet use [12,22,23,24], problematic gaming [25,26], gaming addiction (GA) [27,28], problematic smartphone use [29], and problematic social media use [30] have especially focused on adolescents, young adults, and adults, highlighting that both individual (e.g., male gender, psychopathological symptoms, and personality traits) and contextual factors (e.g., family relationship quality, and peer support) can increase or reduce the risk of developing problematic Internet use [31,32].

On the contrary, only a few studies have explored children’s Problematic Media Use (PMU) [33,34] although researchers and pediatricians have warned about the risks associated with children’s digital media overexposure [35] and shown that early childhood media exposure for a long period may be associated with several negative developmental outcomes, including decreased social and emotional skills [36,37], poor self-regulation abilities [38,39,40], increased attention problems [41,42], sleep problems [43], aggressive behaviors [44,45], and academic difficulties [46]. Therefore, greater attention to PMU in childhood is needed because it may help to identify differences related to specific population (e.g., between children and adolescents) [47], patterns of maladaptive media use preventively and reduce the risk of developing more severe Internet-based addictive behaviors during adolescence and adulthood.

The present study refers to PMU in childhood defining it as excessive use of different screen media devices (e.g., computer, videogames, smartphone, tablet, and television) that interferes with children’s social, behavioral, or academic “functioning” [34,48]. According to Domoff et al. [34], users’ impaired functioning is critical in distinguishing normal/excessive use from a problematic level of media use. Indeed, an excessive amount of time spent using media should not be confused with PMU [34], since there are no convincing results proving that screen time alone directly compromises children’s psychosocial functioning and development [49].

To date, the Interactional Theory of Childhood Problematic Media Use (IT-CPU) [34] represents the only theoretical framework specifically designed to investigate the main risk and protective factors, the underlying psychological processes, and the contextual factors involved in children’s PMU etiology and maintenance. According to the IT-CPU, distal factors (e.g., socioeconomic status, household dysfunction, and digital environment), proximal factors (e.g., general and media-related parenting practices, child behaviors, and peer access to technology) and maintaining factors (e.g., dyadic parent–child relationship, the child’s media use purposes, and peer influence to engage online together) jointly influence the development of PMU in childhood and increase the likelihood of developing a more severe PMU later.

In summary, most studies and literature reviews have explored adolescents’ and young adults’ PMU [20,26,31,50]. Although there have been some literature reviews which analyzed problematic smartphone use [51] or Internet Gaming Disorder [52] in both children and adolescents, they focused only on children with an average age of more than 10 years old. Conversely, few empirical and theoretical studies, and no systematic literature review, have been conducted on PMU among children under the age of 10, because PMU in childhood has gained attention only recently and is still in its infancy [33,34]. Consequently, a wide perspective on the prevalence, manifestation, risk and protective factors linked to children’s PMU is needed urgently, as part of current research has shown that the COVID-19 pandemic has led to an increased use of digital devices in all age groups, and probably also to the possibility of developing risky, problematic, or addictive media use [7,8,53,54], although unanimous consensus has not yet been reached on this point [55]. Therefore, the aim of this systematic review of the studies published in the last 10 years from 2012 to 2022 is to identify risk and protective factors both at individual and contextual levels that might increase or reduce the risk of developing PMU among children aged 0–10 years, as well as the main instruments used to measure children’s PMU. Specifically, the purpose of this review is to answer the following research questions:

1. What individual and contextual risk factors increase PMU among children?
2. What individual and contextual protective factors decrease PMU among children?
3. What negative outcomes are associated with children’s PMU?
4. What are the main screening instruments used to measure PMU among children?

Finally, these findings might be useful to direct future research in this field, as well as to implement programs to prevent maladaptive media use and to promote more functional and healthy media use among children.

## 2. Methods

This systematic review followed the Preferred Reporting Items for Systematic reviews and Meta-Analyses (PRISMA) [56].

### 2.1. Eligibility Criteria

The inclusion criteria for the present review were (i) studies containing quantitative empirical data; (ii) studies published in the last ten years, from 2012 to 2022; (iii) studies providing a full-text article published in English or Italian; (iv) studies with mean sample age between 0 and 10 years; and (v) studies concerning problematic or addictive web-mediated behaviors. Studies that did not meet all the eligibility criteria were excluded.

### 2.2. Search Procedures and Study Selection

**Figure 1:**
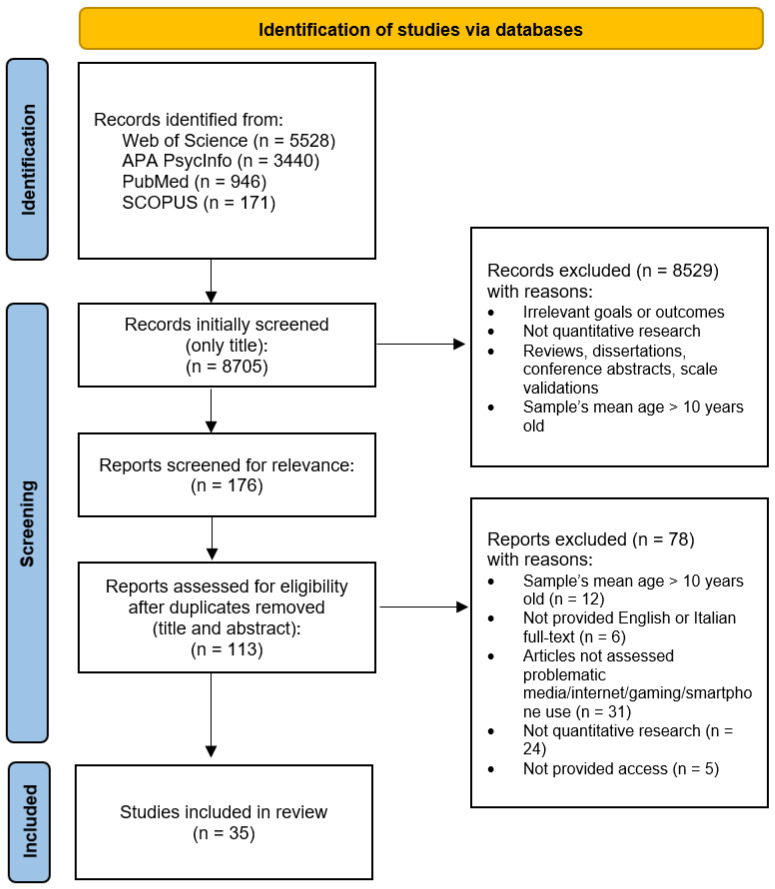
Flow chart of the search strategy and selection procedure.

The literature search was performed in November 2022 across the following electronic databases: Scopus, Web of Science, PsycInfo, Scholar, and PubMed. The following combinations of keywords were entered: “(primary school child* OR preschool child*) AND (problematic internet use* OR problematic gaming use* OR problematic smartphone use* OR problematic media use*)”. The search strategy was represented by a flow diagram (Figure 1). Based on the eligibility criteria, the title and abstract of each article was screened independently by two authors (first and second author) and disagreements were resolved by a third author (third author). Then, the articles that did not meet the inclusion criteria and duplicates were excluded.

### 2.3. Data Extraction and Risk of Biases

Several items of information were extracted, including authors, year of publication, country of data collection, sample size, sample type (e.g., parents or children), mean age and age range (if provided), parents’ gender-related differences, type of problematic/addictive behavior assessed, scales used to measure problematic/addictive behavior, and study design. All included studies were evaluated for risk of bias with the QualSyst tool for evaluating quantitative or qualitative studies [57] (Tables S1–S3). Two authors (first and second author) independently evaluated the selected studies with the standardized QualSyst evaluation tool. In case of disagreement, conflicts were resolved by a third author (third author). The QualSyst tool for quantitative study is a validated generic checklist consisting of 14 items and assessing the scientific validity of reviewed studies. The QualSyst tool evaluated objective (Q1), study design (Q2), subject selection (Q3), subject description (Q4), intervention (Q5, Q6, and Q7), outcome measures (Q8), sample size (Q9), analytic methods (Q10), the estimate of variance (Q11), confounding (Q12), results (Q13), and conclusions (Q14) by assigning a score according to the degree to which the specific criteria were met (‘yes’ = 2, ‘partial’ = 1, ‘no’ = 0). Items not applicable to a particular study design were marked ‘n/a’. In this review, the three items regarding ‘intervention’ were marked as ‘not applicable’. The final summary score was calculated for each paper by summing the total score obtained across the 14 items (i.e., (number of ‘yes’ × 2) + (number of ‘partials’ × 1)) and dividing by the total possible score (i.e., 28—(number of “n/a” × 2)). Overall, the total scores assigned by the first (first author) and the second (second author) reviewer for each quantitative study ranged between 0.60 and 1.00 (mean: 0.86; standard deviation: 0.09), and between 0.55 and 1.00 (mean: 0.84; standard deviation: 0.10), respectively. Reviewers assigned the same total score to 13 articles (37%), while discrepancies for the other 22 studies ranged between 0.04 and 0.14. Studies were included in the systematic review if their summary scores scored ≥ 0.55 (55%), which is considered a relatively liberal threshold [57].

## 3. Results

A total of 10,085 papers were identified from the original search process through electronic databases. Subsequently, 8705 articles were initially screened for only title and 8529 were excluded as they did not meet the inclusion criteria. After the removal of duplicates, 113 full-text articles were screened for title and abstract and assessed for eligibility. Consequently, 78 full-text articles were excluded as they did not meet the inclusion criteria. Finally, 35 studies met the initial inclusion criteria and have been included in the present literature review.

### 3.1. Characteristics of Selected Studies, Prevalence of Children’s PMU, and the Association with Time Usage

As shown in Table 1, most of the selected studies have been published very recently. More specifically, on a total of N = 35 studies, N = 11 studies were published in 2021, N = 7 in 2022, N = 6 in 2020, N = 6 in 2018, N = 2 in 2019, N = 2 in 2017, and finally N = 1 in 2016. Concerning the country of origin, about half of the selected studies (N = 22) were carried out in Eastern countries (mainly in South Korea), N = 9 in Western countries (mainly in the USA), and N = 4 in Middle East countries (only in Turkey). The samples were mainly composed of children rather than parents and most of the latter were mothers. The children’s average age ranged from a minimum of 5.95 months [58] to a maximum of 10.05 years [59], and the samples were well gender-balanced. GA was the most explored online behavior in N = 12 studies, followed by Internet addiction (IA) in N = 7 studies, Problematic Internet Use (PIU) in N = 5 studies, and Problematic Media Use (PMU) in N = 5 studies. Regarding the study design, N = 24 cross-sectional studies and N = 11 longitudinal studies emerged.

Across studies carried out in Eastern countries, the percentage of children classified as potential ‘mobile phone users with problems’ or ‘risky media users’ ranged between 20.9% [60] and 22.91% [61]. Moreover, adolescents showed higher PMU as compared to children. Indeed, the percentage of problematic media users ranged between 3.6% and 6.1% among children aged between 5–9 years old, and between 7.1% and 11% among children aged over 10 years old [62,63]. Furthermore, a study conducted on children from Western countries revealed that 2.5% of boys and 1.4% of girls had PMU, specifically satisfying the Gaming Disorder criteria [64]. During the COVID-19 pandemic, two studies observed a low risk of PMU among children [64,65,66]. In particular, Aközlü et al. [66] reported that 9.7% of the children showed limited PMU symptoms during the COVID-19 pandemic, while 90.3% of the children showed no symptoms. On the contrary, Kroshus et al. [67] highlighted that 32.6% of children between 6 and 10 years had PMU during the COVID-19 pandemic.

A few studies significantly associated the amount of children’s time spent consuming media with PMU [68,69]. Children involved in the ‘high-risk group’ of PMU [61,69,70,71] were found more prone to consume media more than 2 h a day, while those in the ‘no-risk group’ less than 2 h a day [61,69].

### 3.2. Main Instruments Used to Measure Children’s PMU

Concerning screening tools aimed at detecting PMU from a parental perspective, the Problematic Media Use Measure (PMUM) [33] was the most used [58,72,73], followed by the Korean Scale for Internet Addiction (K-Scale) [61,74,75] which was instead found to be the most used screening tool to measure PMU from children’s perspective [61,62,70,75,76,77]. Other employed tools were the Computer Addiction Scale for Children [71,78,79], the Internet Game Use-Elicited Symptom Screen (IGUESS) [80,81,82], the Young Diagnostic Questionnaire for Internet Addiction (YDQ) [10,63,83], and the Chen Internet Addiction Scale (CIAS) [84,85,86]. Self-report measures assessing PMU among children with an average age of 3 and 6 years were mainly completed by parents. On the contrary, in the group with a mean age of 8 and 10 years, self-report measures were mainly completed by children.

### 3.3. Contextual Risk Factors Associated with Children’s PMU

#### 3.3.1. The Role of Family Context: Parent–Child Relationship, Mother’s Psychopathology, Child Maltreatment, and Conflict Situation

Through the studies reviewed, the family context has been found to play a pivotal role in increasing the likelihood of children’s involvement in PMU.

One study showed that dysfunctional parent–child interaction at Time 1 positively predicted a child’s PMU at Time 3 [58]. Moreover, time spent without parents (for 2–4 h a day) was positively associated with higher children’s PMU [61]. Three studies observed that parents experiencing psychological distress during the COVID-19 pandemic [67] and maternal depressive symptoms [58,76] significantly affected PMU among children. More specifically, Holmgren et al. [58] observed that maternal postpartum depression at Time 1 increased the risk of developing PMU in both children and mothers at Time 3. According to Oh et al. [76], the mean PMU score was significantly higher among children with mildly or moderately depressed mothers up to two years after childbirth than among children with non-depressed mothers. Additionally, children with mildly or moderately depressed mothers and higher scores on PMU also experienced more internalizing and externalizing symptoms.

Interestingly, Hsieh et al. [85] showed a significant relationship between post-traumatic stress disorder (PTSD), types of child maltreatment and child’s PMU. Indeed, five types of child abuse, such as psychological neglect, physical neglect, paternal and maternal physical violence, and sexual violence were positively associated with child’s PMU. Finally, parental marital status (such as divorced, separated, or widowed) [70] and lower family income level were associated with children’s higher PMU [69,71,83]. However, in contrast to these results, Abdullah et al. [60] observed that household income level does not significantly contribute to children’s PMU. Other findings demonstrated that various conflict situations in the household contributed to increasing children’s PMU. Maternal work–family conflict was positively associated with children’s PMU, which means that if mothers experienced higher levels of work–family conflict, then children tended to develop higher PMU [70]. Yang et al. [70] highlighted that maternal parenting style mediated the positive association between maternal work–family conflict and children’s PMU, whereby maternal–family conflict contributed to poor maternal parenting, which in turn led to children’s PMU. Jeong et al. [81] observed that parental marital conflict directly and indirectly increased children’s PMU one year later, through poor father–child attachment and low child self-esteem. Specifically, the parental marital conflict led to poor father–child attachment, which in turn decreased child self-esteem, finally leading to higher PMU symptoms.

#### 3.3.2. The Role of Parenting Style and Parental Media Mediation

Concerning the role of parenting style, three studies showed that authoritarian and permissive parenting styles were positively associated with PMU [61,70,86]. Specifically, Lo et al. [87] evidenced that a permissive parenting style significantly moderated the positive association between children’s worrying and PMU. This means that children’s worrying, which is the cognitive process aimed at maintaining a high degree of vigilance about an imminent threat or danger, increases the risk of developing PMU especially when they experienced a permissive parenting style. Furthermore, male gender significantly moderated and amplified the negative association between authoritarian parenting and PMU [86]. In addition, inconsistent parenting practices at Time 1 positively predicted PMU among children at Time 2 [88].

Regarding parental media mediation practices, Van Petegem et al. [89] highlighted that parents who adopted restrictive mediation in a more ‘controlling communication style’ had more negative attitudes towards digital games and perceived more PMU among their children. In line with these findings, Miltuze et al. [88] found that PMU among children was predicted by more forbidding access to the Internet and more technical control (such as using software to block or filtering specific types of content, the amount of time spent online, and the people that child could encounter online). Interestingly, although the absolute number of media-related rules was not associated with children’s PMU, a decline in media-related rules during COVID-19 pandemic as compared to pre-pandemic periods [67], and fewer rules at home regarding Internet use [88] were associated with more PMU. Moreover, Yang et al. [90] pointed out that inconsistent maternal mediation was positively associated with PMU and that this relationship was moderated by two specific parent–child conflict resolution tactics, such as physical assault and psychological aggression. Thus, maternal inconsistent mediation increased the child’s PMU especially when mothers adopted both these negative conflict resolution tactics. In addition, Eales et al. [73] revealed that, during the COVID-19 pandemic, parental perceptions of media as hurting predicted children’s PMU at Time 2.

Coyne et al. [72] observed that media emotion regulation, which is the parents’ tendency to use media to regulate children’s difficult emotions, positively predicted children’s PMU. Additionally, media emotion regulation mediated the relationship between two domains of child temperament (i.e., negative affect and surgency) and the child’s PMU. This means that children with a difficult temperament received media from parents to regulate or avoid problematic emotions, which consequently increased the risk of developing PMU. Moreover, in line with these findings, parents providing mobile devices to settle their children significantly contributed to child PMU [60].

#### 3.3.3. The Role of School Context

Among the studies reviewed, only two studies [63,91] analyzed the role of school context in increasing the PMU. In particular, it was observed that lower school connectedness [91] and school functioning [63] predicted higher PMU among children.

### 3.4. Individual Risk Factors Associated with Children’s PMU

Regarding the individual risk factors that could increase PMU among children, early exposure to mobile devices use [60], higher levels of internalizing and externalizing symptoms [92], executive function problems [75], autistic traits, sensation-seeking [91], depressive symptoms [63,82], anxiety [65], emotional dysregulation [68], post-traumatic stress disorder symptoms (PTSD) [85], previous high levels of PMU [75,88], pre-pandemic PMU [73], and ADHD symptoms [64], significantly predicted higher levels of PMU among children.

Regarding the role of age, studies confirmed that children’s PMU significantly increased with higher age and school grade, and from year to year [63,69,71,73,89,93,94]. Particularly, parents of 3–5-year-old children perceived lower levels of PMU than parents of 6–9-year-old children [89]. In line with this finding, Takahashi et al. [63] noted that the prevalence of PIU and maladaptive Internet use were higher in junior high school children than in elementary school children.

Concerning the gender differences, findings revealed that boys more likely showed PMU than girls [60,61,63,64,65,67,70,71,79,85,86,88,89,91,93,94]. Interestingly, Takahashi et al. [63] showed that gender differences in the prevalence of PMU were related to school grades. Although PMU was higher in 4th and 5th grade boys, this trend was reversed in the 7th grade, whereby PMU became higher in girls.

Findings from the study of Hsieh et al. [86] showed that children’s psychological symptoms were positively associated with PMU and that this relationship was moderated by the male gender. These findings indicated that children’s mental health significantly increased the risk of developing PMU and that this effect was amplified among boys. Regarding the association between internalizing and externalizing symptoms and children’s PMU, Richard et al. [92] highlighted that higher initial levels of these symptoms in childhood significantly predicted PMU six years later in adolescence.

Concerning the relationship between emotion regulation and PMU, children’s emotion dysregulation at Time 1 was positively associated with the time of media usage and higher GA symptoms (only at dimensional level) 5 years later at Time 2 [68], which means that children with emotion dysregulation used media for a longer time and showed higher PMU than children without emotion dysregulation. Moreover, Hsieh et al. [85] showed that PTSD symptoms significantly mediated the association between different types of child maltreatment, such as psychological neglect, physical neglect, paternal physical violence, sexual violence (except maternal physical violence), and PMU. Thus, different child maltreatment experiences increased the risk of developing PTSD, which in turn led to higher levels of PMU. Interestingly, as concerns ADHD symptoms, Paulus et al. [64] observed that the hyperactivity/impulsivity subtype was more important in predicting PMU among boys, while the inattention subtype was more important in predicting PMU among girls. These results aligned with those of Kietglaiwansiri et al. [95], which evidenced that children with ADHD had a higher rate of PMU than control groups. Finally, De Pasquale et al. [65] highlighted that state anxiety, which is a more transient emotional state compared to trait anxiety, was significant in predicting PMU during the COVID-19 pandemic, while trait anxiety was only nearly significant. Moreover, results from Eales et al. [73]’s study revealed that PMU at Time 2 was positively predicted by pre-pandemic PMU at Time 1. In line with this last point, the other two studies proved the longitudinal stability of PMU, by showing that PMU at Time 1 positively predicted PMU at Time 2 [74,88].

### 3.5. Protective Factors Associated with Children’s PMU

Concerning protective factors in the family context, parent–child relationship quality at Time 1 negatively predicted the child’s CIU at Time 2 [88]. Moreover, authoritative parenting style, which involves parents’ exhibiting reasonable emotional control, high responsiveness, and consistency in discipline [61,70,86] and restrictive parental mediation, which involves parents’ setting rules to limit the time of media usage or type of media content that can be accessed on the Internet [88,89,90], were related to lower PMU among children. In line with this result, Song [75] showed that maternal control was higher among children with lower PMU, thus indicating that children whose mothers would monitor their online activities had a lower risk of developing PMU. Moreover, this study pointed out that maternal control negatively predicted executive function problems over the years among children with higher PMU, which means that maternal control on children’s Internet activities reduced executive function problems, especially among children with higher PMU. Finally, some studies observed that a higher maternal educational level (e.g., university degree) was associated with lower PMU among children [61,70,71].

Regarding the individual protective factors that could decrease children’s PMU, it was found that children’s happiness [61] significantly predicted lower PMU. The study by Apisitwasana et al. [59] was the only one aimed at evaluating the effectiveness of the Participatory Learning School and Family Based Intervention Program in preventing children’s GA by developing self-regulation. The findings highlighted that children improved in self-regulation, knowledge, and attitude towards the effect of gaming and GA, while they reported lower scores in GA, immediately and 3 months after the program. These results proved that interventions aimed at improving self-regulation, knowledge, and attitudes about the effects of GA by means of school and family-based participatory learning significantly reduced its prevalence among children.

### 3.6. Negative Outcomes Associated with Children’s PMU

Although previous studies have investigated PMU mainly as an outcome, other studies have explored children’s PMU as a predictor, thus showing how it could lead to several negative consequences at both scholastic and psychological levels. In particular, studies have shown that children’s PMU was associated with more problematic behaviors, lower emotional intelligence [96], higher depressive symptoms [82], and more sleep problems [93]. Furthermore, the study of Zhou et al. [83] was the only one to analyze the relationship between children’s PMU and the achievement of a specific school subject, thus revealing that higher PMU was associated with lower mathematical self-efficacy and mathematical achievement. Moreover, this study pointed out that the relationship between PMU and mathematical self-efficacy was moderated by the teacher–student relationship, thus demonstrating that higher PMU can negatively impact mathematical self-efficacy through a negative teacher–student relationship.

**Table 1:** Summary of the 35 reviewed studies.

Authors	Country	Sample (N)	Child’s Age	Parents’ Sex (% Female)	Type of Problematic/Addictive Behavior	PMU Scales (Child-Report)	PMU Scales (Parent-Report)	Study Design	Main Findings
Abdullah et al. [60]	Malaysia	N = 364 parents	5 years old (51.1% of the sample) 6 years old	NR	Problematic mobile phone use	-	Problematic Mobile Phone Use Scale (PMPUS)—Malaysian version [97]	Cross-sectional	Male gender, earlier exposure to mobile devices, and parents providing mobile devices to make their children sit, significantly increased PMPU. Parents’ educational level, household income, and type of application did not significantly increased PMPU.
Aközlü et al. [66]	Turkey	N = 154 parents	Mean Age = 8.52 ± 1.20 (Range 7–10 years old)	-	Internet addiction	-	The Family–Child Internet Addiction Scale—Turkish version [10]	Cross-sectional	9.7% of the children showed limited IA symptoms during the COVID-19 pandemic, while 90.3% of the children showed no symptoms. Children’s IA tended to increase when parents frequently warned the child about COVID-19 precautions, parents watched news about COVID-19 with their child, and children played video games with parents less frequently.
Apisitwasana et al. [59]	Thailand	N = 310 children	Mean Age = 9.77 ± 0.79 (Intervention Group) Mean Age = 10.05 ± 0.67 (Control Group) (Range 8–12 years old)	-	Game addiction	Game Addiction Screening Test (GAST) [98]	-	Quasi-experimental	The school and family-based participatory learning intervention significantly increased knowledge, attitude, and self-regulation, while reduced GA scores immediately and 3 months after the program.
Bae et al. [61]	South Korea	N = 1078 parents	Range 8–9 years old	-	Media addiction	-	Korean Scale for Internet Addiction (K-Scale) [74]	Cross-sectional	Authoritative parenting style was found to be negatively associated, while authoritarian and permissive parenting positively associated with MA. Children with higher levels of self-esteem and happiness reported lower levels of MA.
Cho et al. [96]	South Korea	N = 303 parents	Age = Younger than 1 year old (5.9%) 2 years old (11.9%) 3 years old (19.5%) 4 years old (21.5%) 5 years old (21.5%) 6 years old (19.8%) (Range 1–6 years old)	93%	Smartphone addiction proneness		Smartphone Addiction Proneness Scale [99]	Cross-sectional	Children with higher SA tendencies manifested more problematic behaviours and less emotional intelligence.
Coyne et al. [72]	USA	N = 269 parents	Mean Age = 29.58 (months old) ± 3.83	N = 256	Problematic media use	-	Problematic Media Use Measure Short Form (PMUM-SF) [33]	Cross-sectional	Media emotion regulation positively predicted children’s PMU. Media emotion regulation significantly mediated the relationship between the child’s temperament (i.e., negative affect and surgency) and child’ PMU.
De Pasquale et al. [65]	Italy	N = 162 children	Mean Age = 9.4 ± 0.7 (Range 8–10 years old)	-	Video game addiction	Videogame Addiction Scale for Children (VASC) (Yılmaz et al., 2017) [100]	-	Cross-sectional	There was a low risk of GA among children during the COVID-19 pandemic. State anxiety significantly predicted GA during the COVID-19 pandemic, while trait anxiety was only nearly significant.
Eales et al. [73]	USA	N = 129 parents	Mean Age = 6.14 ± 2.21 (Range of 2.33–12.75 years old)	N = 127	Problematic media use	-	Problematic Media Use Measure Short Form (PMUM-SF) [33]	Longitudinal	PMU at T1, child age, and parent perceptions of media as hurting their child significantly predicted PMU at T2.
Holmgren et al. [58]	NR	N = 491 mothers	Mean Age = 5.95 (months old) ± 3.61	100%	Problematic media use	-	Problematic Media Use Measure Short Form [33]	Longitudinal	Dysfunctional parent–child interaction and maternal post-partum depression at Time 1 positively predicted child’ PMU at Time 3.
Hsieh et al. [85]	Taiwan	N = 6233 children	School grade = 4th graders	-	Internet addiction	Chen Internet Addiction Scale (CIAS) [84]		Cross-sectional	Child’s IA was positively correlated with PTSD symptoms and experiencing five types of child abuse (e.g., psychological neglect, physical neglect, paternal and maternal physical violence, and sexual violence). PTSD significantly mediated the association between different types of child maltreatment (except maternal physical violence) and IA.
Hsieh et al. [86]	Taiwan	N = 6233 children	School grade = 4th graders	-	Internet addiction	Chen Internet Addiction Scale (CIAS) [84]	-	Cross-sectional	Authoritative parenting style was found to be negatively associated, while authoritarian and permissive positively associated with IA. Psychological symptoms were positively associated with IA. These associations were enhanced among boys.
Jeong et al. [82]	Korea	N = 366 children	Median Age = 10 years old (Range 9–12 years old)	-	Internet Gaming Disorder	Internet Game Use-Elicited Symptom Screen (IGUESS) [80]	-	Longitudinal	There was a reciprocal causality between the risk of developing GA and the higher levels of depressive symptoms. The level of depression at Time 1 was stronger in predicting the severity of GA one year later at Time 2.
Jeong et al. [81]	Korea	N = 268 children	Mean Age = 9.4 ± 0.6 (Range 9–10 years old)	-	Internet Gaming Disorder	Internet Game Use-Elicited Symptom Screen (IGUESS) [80]	-	Longitudinal	Parental marital conflict caused poor father–child attachment, which in turn decreased child’ self-esteem, finally leading to higher GA symptoms. It was found a reciprocal causality between the risk of developing GA and the higher levels of depressive symptoms. However, the level of depression at Time 1 was stronger in predicting the severity of GA one year later at Time 2.
Kietglaiwansiri et al. [95]	Thailand	N = 80 children (ADHD Group) N = 102 children (Control Group)	Mean Age = 9.5 years old (Range 6–19 years old) (ADHD Group) Mean Age = 10 years old (Range 6–13 years old) (Control Group)	-	Game addiction/problematic video game use	-	Game Addiction Screening Test (GAST) [98]	Cross-sectional	Children with ADHD symptoms (e.g., inattention and hyperactivity/impulsivity) reported higher levels of GA than controls.
Kök Eren and Örsal [79]	Turkey	N = 205 children	School grade = 4th graders (Range 9–10 years old)	-	Computer game addiction	Computer Game Addiction Scale [78]	-	Descriptive	There was a positive association between child’s loneliness and GA.
Kroshus et al. [67]	USA	N = 547 parents	Range of 6–10 years old		Problematic media use	-	Problematic Media Use Measure Short Form (PMUM-SF) [33]	Cross-sectional	During COVID-19 pandemic, problematic media use was higher when parents were employed full time, present in the home, had low/formal educational attainment, and more psychological distress. There was no significant association between rule implementation and problematic media use.
Lim et al. [62]	Korea	N = 1221 children	Range of 5–9 years old	-	Problematic Internet use	-	Korean Scale for Internet Addiction (K-scale) [101]	Cross-sectional	Level of PIU was higher in adolescents than in children. Children exhibited higher depressive symptoms when they had more severe PIU.
Liu et al. [91]	China	N = 420 children	Mean Age = 9.74 ± 0.45	-	Internet gaming addiction	Pathological Video Game Use Questionnaire [102]		Longitudinal	Autistic traits at Time 1 predicted lower emotion regulation at Time 2, which in turn predicted lower school connectedness at Time 3, which in turn predicted higher GA at Time 4.
Lo et al. [87]	China	N = 227 children	Mean Age = 9.55 ± 0.58 (Range of 8–12 years old)	-	Internet addiction	Internet Addiction Test (IAT) [10]	-	Cross-sectional	Permissive parenting style significantly moderated the positive association between children’s worrying and IA
Miltuze et al. [88]	Latvia	N = 261 children (Time 1) N = 236 children (Time 2)	Mean Age = 8.55 ± 2.02 (Time 1) Mean Age = 9.79 ± 1.47 (Time 2) (Range 8–11 years old)	87%	Compulsive Internet use	Compulsive Internet Use Scale [103]	-	Longitudinal	Parent–child relationship quality at Time 1 negatively predicted child’ CIU at Time 2. Inconsistent parenting practices, less rules at home regarding Internet use, more forbidding access to the Internet, and more technical control at Time 1 positively predicted CIU in children at Time 2.
Muslu et al. [71]	Turkey	N = 476 children	Age = 8 years old (N = 87) 9 years old (*n* = 226) 10 years old (*n* = 163)	-	Computer game addiction	Computer Addiction Scale for Children [78]		Descriptive	Boys were more prone than girls to have GA. GA increased significantly with higher age and school grade Lower maternal educational level and lower family income level were associated with higher GA.
Oh et al. [76]	South Korea	N = 1132 children	Age = 9 years old	100%	Problematic Internet use	Korean Internet Addiction Scale (K-scale) [104]	-	Longitudinal	PIU score was significantly higher in children with mildly or moderately depressed mothers. Children with mildly or moderately depressed mother and higher score on PIU also experienced more internalizing and externalizing symptoms.
Oh et al. [77]	South Korea	N = 1389 children	Age = 9 years old	-	Problematic media device use	Korean Internet Addiction Scale (K-scale) [104]	-	Cross-sectional	Children at higher risk of PMU exhibited more internalizing and externalizing behavior problems, as well as suicidal ideation and suicidal behavior.
Park et al. [69]	South Korea	N = 1378 children	Mean Age = 4.6 ± 1.11 (Non-PSU group) Mean Age = 4.8 ± 1.06 (PSU group)	-	Problematic smartphone use	-	Korean-language Smartphone Overdependence Scale (S-scale) for children [105]	Cross-sectional	Time spent consuming media was significantly associated with children’s PSU. PMU significantly increased with higher age and lower household income.
Paulus et al. [64]	Germany	N = 1271 children	Mean Age = 5.8 ± 0.38 (Range of 4.4–8.2 years old)	-	Computer gaming disorder	-	Young Children-Computer Gaming Disorder (YCCGD) (ad hoc questionnaire based on the substance-related addiction criteria of ICD-10)	Cross-sectional	Children with ADHD symptoms reported higher levels of GA.
Paulus et al. [68]	Germany	N = 80 parents	Mean Age = 4.2 ± 1.23 (Time 1) Mean Age = 9.2 ± 2.03) (Time 2)	-	Gaming Disorder	-	The 9-item questionnaire was based on the DSM-5 criteria relating to IGD [106]	Quasi-experimental	Children’s emotion dysregulation at Time 1 was positively associated with time of media usage and higher GA symptoms (only at the dimensional level) 5 years later at Time 2.
Richard et al. [92]	France	N = 744 children	Mean Age = 8.3 ± 0.93 (Range 6.3–9.9 years old at Time 1)	NR	Internet Gaming Disorder Problematic video gaming	The Internet gaming disorder (IGD) criteria out- lined in Section III of the DSM-5 [16]	-	Longitudinal	Higher levels of internalizing and externalizing symptoms in childhood significantly predicted GA 6 years later in adolescence.
Sakamoto et al. [93]	Japan	N = 6893 children	Mean Age = 9.0 ± 1.8 (Range 6–12 years old)	NR	Internet Addiction	-	Ad hoc questionnaire based on Young Diagnostic Questionnaire for Internet Addiction (YDQ) [10]	Cross-sectional	Boys were more prone than girls to have IA. IA was positively associated with children’ sleep problems. IA was associated with higher age and school grade.
Sayı et al. [94]	Turkey	N = 157 children N = 16 teachers	Age = 8 years old (N = 55) 9 years old (N = 61) 10 and above years old (N = 114) (Range 8–12 years old)	-	Internet and Gaming addiction	Computer Addiction Scale for Adolescents [107]	-	Descriptive	School social competence (including interpersonal skills, self-management skills and academic skills) negatively correlated with both IA and GA among gifted children.
Song [75]	South Korea	N = 1463 mothers	8 years old at Wave 9 9 years old at Wave 10 10 years old at Wave 11	100%	Children Internet Addiction Status	-	Korean Diagnostic Scale for Internet Overdependence (K-scale) [74]	Longitudinal	Maternal control was higher among children in the low-risk IA group. Children at higher risk of IA reported more executive function problems.
Takahashi et al. [63]	Japan	N = 3845 children	Age/School grade = 4th grade (Range 9–10 years old) 5th grade (Range 10–11 years old) 6th grade (Range of 11–12 years old)	-	Problematic Internet use	Young’s Diagnostic Questionnaire (YDQ) [10]	-	Cross-sectional	PIU was higher in junior high school children than in elementary school children. PIU was higher in 4th and 5th grade boys, while PIU became higher in 7th grade girls. Children exhibited higher depressive symptoms when they had more severe PIU. Children with PIU exhibited lower health-related quality of life.
Van Petegem et al. [89]	NR	N = 762 parents	Mean Age = 5.52 ± 1.86 (Range of 3–9 years old)	82.6% females	Problematic gaming	-	Video Game Addiction Test (VAT) [108]	Cross-sectional	Restrictive mediation was related to less PGU. Parents who adopted restrictive mediation in a more controlling style tended to perceive more PGU among their children. Preschool children had lower PMU levels than primary school children.
Yang et al. [70]	South Korea	N = 707 Children	Mean Age = 9.4 ± 0.12 (Range 9–10 years old)	100%	Problematic Internet use	Korean Internet addiction scale (K-scale) for adolescents [74]	-	Cross-sectional	Authoritative parenting style was negatively associated, while authoritarian and permissive positively associated with PIU. Maternal parenting style moderated the positive association between maternal work–family conflict and children’s PIU.
Yang et al. [90]	Singapore	N = 154 mothers	Mean Age = 61.42 (months old) ± 8.93 (Range 42–77 months old)	100%	Problematic smartphone use	-	Smartphone Addiction Scale (Short Form) adapted for children [109]	Cross-sectional	Restrictive mediation was associated with lower PSU. Maternal inconsistent mediation was positively associated with PSU, and this relationship was moderated by specific parent–child conflict resolution tactics (e.g., physical assault and psychological aggression)
Zhou et al. [83]	China	N = 4300 children	Age = 10 years old	-	Problematic Internet use	Young Diagnostic Questionnaire for Internet Addiction (YDQ) [10]		Cross-sectional	Higher PIU predicted lower mathematics achievement. The association between PIU and maths achievement was mediated by mathematical self-efficacy, and the relationship between PIU and mathematical self-efficacy was mediated by teacher–student relationship.

## 4. Discussion

The present literature review aimed to explore the main risk and protective factors, the measurement instruments and the negative consequences associated with PMU among children aged between 0 and 10 years.

In terms of cultural differences, it was found that the percentage of children classified as “problematic media users” was slightly higher in Eastern countries [62,63] than in Western countries [64]. This result is in line with the systematic review by Dahl and Bergmark [110], according to which prevalence and persistence estimates of PIU were generally higher in Asian countries. Specifically, Bae et al. [61] observed that Korean children showed the highest prevalence of PMU when compared to other Asian countries and to Europe overall. This could be explained by the fact that Korean Information Technology (IT) infrastructures are particularly advanced, and children can use digital devices with free and high-speed connections in many public places at any time [111]. Therefore, it is likely that distal factors, such as cultural aspects, norms, lifestyles, and advanced digital environments affect the risk of children’s PMU [110]. However, it is not possible to draw conclusions on the higher prevalence of PMU in Eastern children compared to Western children due to the disproportion between studies conducted on Eastern (N = 22 studies) and Western samples (N = 9 studies).

Interestingly, several reviewed studies (N = 11 studies) have been published in 2021. Likely, the COVID-19 pandemic has raised greater concerns over the Internet-related risks. Since there has been greater use of the Internet by even younger children both to access distance learning and to communicate or play with their friends, several studies have begun to focus more attention on PMU in this age group as well [112]. Although some studies have shown a significant increase in the time spent on the Internet during the COVID-19 pandemic [8], PMU prevalence estimates have differed widely across countries [55]. In this review, the studies of De Pasquale et al. [65] and Aközlü et al. [66] reported low levels of PMU symptoms among children during the COVID-19 pandemic. These results seem to align with those of Burkauskas et al. [55], according to which increased time spent online per se did not lead to PMU. However, Kroshus et al. [67] observed that 32.6% of children manifested PMU, and that symptoms were higher in families where parents experienced more psychological distress. Therefore, the severity of PMU symptoms during the COVID-19 pandemic might be determined by other underlying motivations, purposes, contextual and psychological conditions, such as higher state anxiety [65], parent’s distress [67], or pre-pandemic PMU symptoms [73].

Among the problematic media-based behaviors, GA has been the most explored addictive behavior in many reviewed studies, probably because it has recently emerged as a global problem included in the appendix of the 5th edition of Diagnostic and Statistical Manual of Mental Disorder (DSM-5) [16] and in the 11th final revision of the International Classification of Diseases (ICD-11) [17]. Moreover, this interest in children’s GA could be explained by the fact that healthy play has always represented one of the most rewarding activities for children by promoting the development of social, emotional, self-regulatory, and cognitive skills, together with creative thinking [113]. However, as digital play has largely replaced material play, research has begun to focus not only on the positive effects but also on the negative effects associated with the use of digital games. For example, recent studies have shown that digital games inhibited children’s use of self-regulatory private speech during problem-solving activities [40], and increased children’s anti-social behaviors and isolation [114]. To date, videogaming represents one of the main sources of entertainment for children [1]. For this reason, several studies examined in this review have attempted to focus especially on excessive videogame use to understand how this might lead to addictive tendencies in children.

Studies have confirmed that time spent consuming media (on average more than 2 h per day) represents an important risk factor that could increase the likelihood of developing PMU among children [61,68,69,70,71]. These data supported the recommendations of the World Health Organization [115], which suggested that the total sedentary screen time for children up to 3–4 years old should not exceed one hour per day, while for children less than one year old should not be encouraged at all. Moreover, the American Academy of Pediatrics [116] also recommended limiting children’s total screen media use to 2 h or less per day. However, it is important to note that impaired psychological functioning and development is not necessarily linked only to screen time, but also to the interaction of other distal, proximal, or maintaining factors, which together can increase the risk of developing PMU and lead to negative consequences for psychological health [34,49].

Regarding the main screening tools, the Problematic Media Use Measure (PMUM) [33] has been the most used parent-report instrument aimed at measuring PMU among children with an average age of 3 and 6 years. On the other end, the Korean Scale for Internet Addiction (K-Scale) [74] was the most used self-report instrument aimed at measuring PMU both from parents and children’s perspectives, especially among children with an average age of 8 and 10 years. These findings confirmed that there are still few validated and reliable screening tools used to detect PMU among children under the age of 10, thus reflecting the limited advancement of research in this field. Of these two instruments, only the PMUM [33] was specifically designed to detect parent’s perspectives regarding PMU among children aged between 4 and 11 years. Instead, as observed by Mak et al. [117], the K-Scale [74] was first developed for adolescents, and later adapted for children and adults as well. Although in the literature there are other tools specifically designed for children aged 6 to 12 and aimed at measuring problematic gaming use, GA, and digital addiction, the related measures (VGS-C, VASC, and DASC, respectively) [100,117,118] have been less used across the reviewed studies. Indeed, only the VASC [100] was used in one study in this review to measure GA in a sample of 9-year-old children. These results have informed us that screening tools specifically designed to assess children’s personal perspectives and representation of their PMU are still few and far between and underused.

In terms of gender-related differences, most of the reviewed studies revealed that boys were more likely to manifest PMU than girls [60,61,63,64,65,67,70,71,79,85,86,88,89,91,93,94], and that this trend was reversed in junior high school, whereby PMU became higher in girls [63]. Likely, males and females are involved in different web-mediated activities for different gender-specific motivations depending on the developmental stage they are going through. In fact, some studies have shown that female adolescents were more likely to use social networking sites, as they represented means of nurturing relationships with members of their peer group [119,120], and to be involved in problematic social network sites use [30,121] when compared to boys and younger children. On the contrary, male children preferred to play video games and were more exposed to problematic gaming use than females [122,123,124].

Studies have observed that PMU increased significantly with higher age and school grade [63,69,71,73,89,93,94]. Therefore, preschool children had lower PMU levels than primary school children [89], while the latter had lower PMU levels than junior high school children [63]. Likely, as they entered pre-adolescence, children acted more independently and consequently their media use was less monitored by parents, thus increasing the risk of developing patterns of problematic use.

As in a recent review focused on adolescent and young adult samples [31], this review highlighted the importance of focusing attention on the risk and protective factors at both individual and contextual level associated with children’s PMU since it might lead to several negative consequences for their health and psychosocial well-being [82,83,93,96]. In line with previous studies which proved the pivotal role of the quality of the family environment and parent–child relationship in the development and maintenance of various problematic and addictive behaviors in adolescence and young adulthood [125,126,127,128], findings of the present review have highlighted that children’s PMU might be often fostered by a dysfunctional family context and adverse parent–child relational experiences [58,67,70,76,81,85], and worse individual psychological symptoms [63,64,65,68,75,82,85,88,91,92]. Probably, as seen in other studies based on samples of adults, young adults, and adolescents [129,130,131,132,133,134,135], all these stressful situations and conditions within the family system may lead to children’s insecure attachment [136,137], which could elicit emotional distress and lead children to experience internalizing and externalizing symptoms that they try to manage by escaping in the virtual environments, thus increasing the risk of developing PMU. Therefore, according to the Compensatory Internet Use Theory [138], PMU might represent a maladaptive coping strategy aimed at both compensating for unmet relational and emotional needs and relieving psychological suffering. In this regard, the study of Coyne et al. [72] observed that higher PMU was associated with media emotion regulation, which is the parent’s tendency to give a digital device (e.g., a smartphone, or a tablet) to their children to manage their difficult emotions, especially if children had a difficult temperament. It is well-known that children learn emotional regulation skills within early parent–child relationships [139,140,141], and that parents with greater emotional dysregulation also show poor reflective functioning, namely a reduced capacity to recognize their infant’s mental states and behaviors [142,143]. Therefore, if media emotion regulation becomes part of everyday interpersonal dynamics, then the child’s opportunities to develop more adaptive emotion regulation strategies and self-regulation skills may be inhibited [144]. Moreover, if parents are prone to use media as a calming tool for their children’s strong emotions, then their capacity for self-regulation and mentalizing may also be weakened, leading to consequent higher difficulties in managing potential future problematic behaviors [34]. Conversely, when parents relied upon a supportive, warmth, and responsive parenting style and set clear rules and limits on media usage without overdoing their controlling style [61,70,75,86,87,88,89,90], they were more likely to have a positive relationship with their children, which provides the basis for positive development and lower PMU among children. As observed in previous studies [145,146,147], parents should adopt a more authoritative parenting style, active parental mediation strategies, and consistent parenting practices, especially during the transition from childhood to adolescence, because in this phase children develop new self-regulation skills [148] and seek greater autonomy from parental figures by passing through the second separation–individuation process [149]. Therefore, an educational and media-mediation style that is too controlling and restrictive may be perceived as an intrusion into the adolescent’s personal autonomy, thus leading to higher PMU [150]. Instead, it has been found that parental mediation strategies, even of a restrictive type, significantly reduced the risk of developing problematic web-mediated behaviors, especially in younger children [151].

Finally, some studies have observed that even the school context, through poor school connectedness [91] and school functioning [63], might increase PMU among children. The school- and family-based intervention of Apisitwasana et al. [59] has been shown to be effective in reducing PMU among children by improving self-regulation skills. According to previous studies [152,153], self-regulation abilities played a crucial role in preventing PMU. Moreover, considering that these self-regulation skills begin to develop during infancy and then during the transition to adolescence [148], and that parents and teachers play a fundamental role in helping children to develop self-regulatory skills [154], it is important to encourage collaboration between schools and families to implement future effective prevention interventions for reducing PMU among children.

### Limitations and Future Research Directions

Some limitations of the examined studies and future research directions should be addressed. Firstly, most of the studies were based on self-report measures, making the results susceptible to answer accuracy and social desirability. To avoid this bias, self-report measures should be supported by more qualitative methods (i.e., observational studies, in-depth interviews, and focus-group discussions). Secondly, self-report instruments specifically designed to get the perspective of children aged 6–10 years were few and not so widely used. Indeed, most of the scales administered to children were originally developed for adolescents and adults and then adapted for children. Since parent and child perspectives on PMU can be very different, future studies should integrate both specific parent- and child-based measures and rely more on quali-quantitative methods to provide a more comprehensive view of the different representations of children’s PMU. Thirdly, some measurement scales were aimed at detecting the presence or absence of ‘addictive’ or ‘problematic’ media behavior among children by adopting a more categorical and less dimensional approach. However, future research should rely more on instruments aimed at identifying and distinguishing between different levels of media engagement (e.g., problematic, risky, or non-problematic). As noted by Paulus et al. [68], a dimensional approach allows the clinician greater flexibility in assessing the severity of a condition without scoring a threshold between normality and disturbance, which is extremely important when assessing the presence or absence of problematic conditions in childhood. Fourthly, since childhood is characterized by numerous developmental changes, future studies should pay more attention to how PMU dynamically evolves through the different stages of childhood development (i.e., early, mid, or late childhood). Fifthly, as parenting practices can vary across Eastern and Western countries [155], future studies should explore whether cultural aspects differently influence the association between parenting style, parental media mediation and children’s PMU. Sixthly, as most of the studies are cross-sectional, additional longitudinal studies are needed to more firmly support the causal relationship between the variables involved. Finally, most of the reviewed studies were based only on maternal samples and few studies have highlighted the role of parental gender differences in the development of PMU. Since Jeong et al. [81] pointed out that father–child attachment had a more important mediating role than mother–child attachment in the association between marital conflict and GA symptoms among children, further studies should look more carefully at how the quality of maternal and paternal relationship may differently decrease or increase the risk of developing PMU among children.

Moreover, this systematic review has some limitations. Firstly, all the reviewed studies explored the relationship between children and various devices and/or web-mediated activities, such as videogame, smartphone, Internet, and media use. For practical reasons, we adopted the label “Problematic Media Use” (PMU) to refer to all these different behaviors or devices. In fact, studies related to each web-mediated activity and device were few and it was not possible to draw meaningful conclusions on the different problematic behaviors. Therefore, further review studies should explore the problematic use of specific devices or web-mediated behaviors in childhood. Secondly, some unindexed papers in the relevant databases may have been unwittingly missed out during the original search process. Thirdly, as research on children’s PMU is still in its infancy, only studies conducted between 2012 and 2022 were included in this review. However, it is possible that some relevant studies published before 2012 have been excluded. Finally, some interesting studies may have been excluded during the original research process because they were not written in English or in Italian, or because the different terms used in the literature to refer to the PMU made it difficult to track them.

## 5. Conclusions

This review has shown that this research field is still in its infancy. Indeed, most of the studies aimed at exploring PMU among children were published in 2021 and carried out in Eastern countries, which means that it needs to be further explored in future studies. Both parent- and child-report instruments were used to assess children’s PMU. However, self-report measures specifically designed to assess PMU from the child’s perspective are still few and far between and not so widely used. Indeed, the most used child-report instruments among reviewed studies were first developed for adolescents or adults, and then adapted for children.

Although the increased access to the internet by even younger children during the COVID-19 pandemic has raised some concerns, the risk of developing PMU did not increase among children. It was probably not an excessive amount of time spent consuming media per se, but concomitant or pre-existing psychological conditions that increased the risk of PMU among children during the COVID-19 pandemic.

GA was the most explored problematic behavior among children. Studies highlighted that using media more than 2 h a day, male gender, and higher age significantly increased the risk of developing PMU. Overall, both individual and contextual factors can increase children’s PMU. Indeed, children who experienced negative psychological symptoms, dysfunctional family environment and lower school functioning were more likely to develop PMU. Moreover, children’s PMU was found to be associated with several negative consequences for their psychosocial well-being. However, authoritative parenting style and restrictive parental mediation decreased PMU among children.

Future prevention interventions should promote better self-regulation skills among children through active collaboration between school and family. Furthermore, since children’s self-regulation skills primarily develop within the parent–child relationship, future interventions should also be aimed at helping parents to improve their self-regulatory and mentalizing capacities, as well as their parental mediation strategies and general parenting practices.

## Data Availability

The data that support the findings of this study are available via email from the corresponding author upon reasonable request.

## Supplementary Materials

The following supporting information can be downloaded at: https://www.mdpi.com/article/10.3390/ijerph20105854/s1, Table S1: Quality assessment of the reviewed studies using the QUALSYST evaluation tool (Author 1); Table S2: Quality assessment of the reviewed studies using the QUALSYST evaluation tool (Author 2); Table S3: Inter-rater agreement and disagreement for overall scores of quantitative studies.

## References

[1] Smahel D., MacHackova H., Mascheroni G., Dedkova L., Staksrud E., Olafsson K., Hasebrink U. (2020). EU Kids Online 2020: Survey Results from 19 Countries.

[2] Livingstone S., Haddon L., Görzig A., Ólafsson K. (2010). Risks and Safety on the Internet: The Perspective of European Children: Full Findings and Policy Implications from the EU Kids Online Survey of 9–16 Year Olds and Their Parents in 25 Countries.

[3] Rideout V., Robb M.B. (2020). The Common Sense Census: Media Use by Kids Age Zero to Eight.

[4] OFCOM U.K. (2021). Children and Parents: Media Use and Attitudes Report.

[5] Duan L., Shao X., Wang Y., Huang Y., Miao J., Yang X., Zhu G. (2020). An investigation of mental health status of children and adolescents in china during the outbreak of COVID-19. J. Affect. Disord..

[6] Ellis W.E., Dumas T.M., Forbes L.M. (2020). Physically isolated but socially connected: Psychological adjustment and stress among adolescents during the initial COVID-19 crisis. Can. J. Behav. Sci. Rev. Can. Sci. Comport..

[7] Oka T., Hamamura T., Miyake Y., Kobayashi N., Honjo M., Kawato M., Kubo T., Chiba T. (2021). Prevalence and risk factors of internet gaming disorder and problematic internet use before and during the COVID-19 pandemic: A large online survey of Japanese adults. J. Psychiatr. Res..

[8] Masaeli N., Farhadi H. (2021). Prevalence of Internet-based addictive behaviors during COVID-19 pandemic: A systematic review. J. Addict. Dis..

[9] Young K.S. (1996). Psychology of Computer Use: XL. Addictive Use of the Internet: A Case That Breaks the Stereotype. Psychol. Rep..

[10] Young K.S. (1998). Internet addiction: The emergence of a new clinical disorder. Cyberpsychol. Behav..

[11] Widyanto L., Griffiths M. (2006). ‘Internet addiction’: A critical review. Int. J. Ment. Health Addict..

[12] Caplan S.E. (2002). Problematic Internet use and psychosocial well-being: Development of a theory-based cognitive–behavioral measurement instrument. Comput. Hum. Behav..

[13] Davis R.A. (2001). A cognitive-behavioral model of pathological Internet use. Comput. Hum. Behav..

[14] Ferrante L., Venuleo C. (2021). Problematic Internet Use among adolescents and young adults: A systematic review of scholars’ conceptualisations after the publication of DSM5. Mediterr. J. Clin. Psychol..

[15] Moretta T., Buodo G., Demetrovics Z., Potenza M.N. (2022). Tracing 20 years of research on problematic use of the internet and social media: Theoretical models, assessment tools, and an agenda for future work. Compr. Psychiatry.

[16] American Psychiatric Association (2013). Diagnostic and Statistical Manual of Mental Disorders (DSM-5).

[17] World Health Organization (2018). The ICD-11 Classification of Mental and Behavioral Disorders: Diagnostic Criteria for Research.

[18] Young K.S., Rogers R.C. (1998). The relationship between depression and Internet addiction. Cyberpsychol. Behav..

[19] Kuss D.J., Van Rooij A.J., Shorter G.W., Griffiths M.D., van de Mheen D. (2013). Internet addiction in adolescents: Prevalence and risk factors. Comput. Hum. Behav..

[20] Kuss D.J., Griffiths M., Karila L., Billieux J. (2014). Internet addiction: A systematic review of epidemiological research for the last decade. Curr. Pharm. Des..

[21] Gioia F., Boursier V., Bozoglan B. (2019). Treatment of Internet addiction and Internet gaming disorder in adolescence: A systematic review. Advances in Psychology, Mental Health, and Behavioral Studies (APMHBS) Book Series. Multifaceted Approach to Digital Addiction and Its Treatment.

[22] Caplan S.E. (2003). Preference for online social interaction: A theory of problematic Internet use and psychosocial well-being. Commun. Res..

[23] Caplan S.E. (2010). Theory and measurement of generalized problematic Internet use: A two-step approach. Comput. Hum. Behav..

[24] Kormas G., Critselis E., Janikian M., Kafetzis D., Tsitsika A. (2011). Risk factors and psychosocial characteristics of potential problematic and problematic internet use among adolescents: A cross-sectional study. BMC Public Health.

[25] Haagsma M.C., Caplan S.E., Peters O., Pieterse M.E. (2013). A cognitive-behavioral model of problematic online gaming in adolescents aged 12–22 years. Comput. Hum. Behav..

[26] Festl R., Scharkow M., Quandt T. (2013). Problematic computer game use among adolescents, younger and older adults. Addiction.

[27] Bonnaire C., Baptista D. (2019). Internet gaming disorder in male and female young adults: The role of alexithymia, depression, anxiety and gaming type. Psychiatry Res..

[28] Gioia F., Colella G.M., Boursier V. (2022). Evidence on Problematic Online Gaming and Social Anxiety over the Past Ten Years: A Systematic Literature Review. Curr. Addict. Rep..

[29] Casale S., Fioravanti G., Benucci S.B., Falone A., Ricca V., Rotella F. (2022). A meta-analysis on the association between self-esteem and problematic smartphone use. Comput. Hum. Behav..

[30] Boursier V., Gioia F., Griffiths M.D. (2020). Do selfie-expectancies and social appearance anxiety predict adolescents’ problematic social media use?. Comput. Hum. Behav..

[31] Anderson E.L., Steen E., Stavropoulos V. (2017). Internet use and problematic internet use: A systematic review of longitudinal research trends in adolescence and emergent adulthood. Int. J. Adolesc. Youth.

[32] Nielsen P., Favez N., Rigter H. (2020). Parental and family factors associated with problematic gaming and problematic internet use in adolescents: A systematic literature review. Curr. Addict. Rep..

[33] Domoff S.E., Harrison K., Gearhardt A.N., Gentile D.A., Lumeng J.C., Miller A.L. (2019). Development and validation of the problematic media use measure: A parent report measure of screen media “addiction” in children. Psychol. Popul. Media Cult..

[34] Domoff S.E., Borgen A.L., Radesky J.S. (2020). Interactional theory of childhood problematic media use. Hum. Behav. Emerg. Technol..

[35] Ferrara P., Corsello G., Ianniello F., Sbordone A., Ehrich J., Giardino I., Pettoello-Mantovani M. (2017). Internet Addiction: Starting the Debate on Health and Well-Being of Children Overexposed to Digital Media. J. Pediatr..

[36] Hinkley T., Brown H., Carson V., Teychenne M. (2018). Cross sectional associations of screen time and outdoor play with social skills in preschool children. PLoS ONE.

[37] Mundy L.K., Canterford L., Olds T., Allen N.B., Patton G.C. (2017). The association between electronic media and emotional and behavioral problems in late childhood. Acad. Pediatr..

[38] Radesky J.S., Silverstein M., Zuckerman B., Christakis D.A. (2014). Infant self-regulation and early childhood media exposure. Pediatrics.

[39] Inoue S., Yorifuji T., Kato T., Sanada S., Doi H., Kawachi I. (2016). Children’s media use and self-regulation behavior: Longitudinal associations in a nationwide Japanese study. Matern. Child Health J..

[40] Bochicchio V., Keith K., Montero I., Scandurra C., Winsler A. (2022). Digital media inhibit self-regulatory private speech use in preschool children: The “digital bubble effect”. Cogn. Dev..

[41] Christakis D.A., Zimmerman F.J., Di Giuseppe D.L., McCarty C.A. (2004). Early television exposure and subsequent attentional problems in children. Pediatrics.

[42] Swing E.L., Gentile D.A., Anderson C.A., Walsh D.A. (2010). Television and video game exposure and the development of attention problems. Pediatrics.

[43] Garrison M.M., Liekweg K., Christakis D.A. (2011). Media use and child sleep: The impact of content, timing, and environment. Pediatrics.

[44] Robinson T.N., Wilde M.L., Navracruz L.C., Haydel K.F., Varady A. (2001). Effects of reducing children’s television and video game use on aggressive behavior: A randomized controlled trial. Arch. Pediatr. Adolesc. Med..

[45] Manganello J.A., Taylor C.A. (2009). Television exposure as a risk factor for aggressive behavior among 3-year-old children. Arch. Pediatr. Adolesc. Med..

[46] Adelantado-Renau M., Moliner-Urdiales D., Cavero-Redondo I., Beltran-Valls M.R., Martínez-Vizcaíno V., Álvarez-Bueno C. (2019). Association between screen media use and academic performance among children and adolescents: A systematic review and meta-analysis. JAMA Pediatr..

[47] Beard K.W., Yarnall C.B. (2008). Internet addiction in children and adolescents. Computer Science Research Trends.

[48] Domoff S.E., Borgen A.L., Foley R.P., Maffett A. (2019). Excessive use of mobile devices and children’s physical health. Hum. Behav. Emerg. Technol..

[49] Ophir Y., Rosenberg H., Tikochinski R. (2021). What are the psychological impacts of children’s screen use? A critical review and meta-analysis of the literature underlying the World Health Organization guidelines. Comput. Hum. Behav..

[50] Lozano-Blasco R., Robres A.Q., Sánchez A.S. (2022). Internet addiction in young adults: A meta-analysis and systematic review. Comput. Hum. Behav..

[51] Fischer-Grote L., Kothgassner O.D., Felnhofer A. (2019). Risk factors for problematic smartphone use in children and adolescents: A review of existing literature. Neuropsychiatrie.

[52] Paulus F.W., Ohmann S., Von Gontard A., Popow C. (2018). Internet gaming disorder in children and adolescents: A systematic review. Dev. Med. Child Neurol..

[53] Király O., Potenza M.N., Stein D.J., King D.L., Hodgins D.C., Saunders J.B., Grif M.D., Gjoneska B., Billieux J., Brand M. (2020). Preventing problematic internet use during the COVID-19 pandemic: Consensus guidance. Compr. Psychiatry.

[54] Sun Y., Li Y., Bao Y., Meng S., Sun Y., Schumann G., Kosten T., Strang J., Lu L., Shi J. (2020). Brief Report: Increased Addictive Internet and Substance Use Behavior During the COVID-19 Pandemic in China. Am. J. Addict..

[55] Burkauskas J., Gecaite-Stonciene J., Demetrovics Z., Griffiths M.D., Király O. (2022). Prevalence of problematic internet use during the COVID-19 pandemic. Curr. Opin. Behav. Sci..

[56] Page M.J., McKenzie J.E., Bossuyt P.M., Boutron I., Hoffmann T.C., Mulrow C.D., Shamseer L., Tetzlaff J.M., Akl E.A., Brennan S.E. (2021). The PRISMA 2020 statement: An updated guideline for reporting systematic reviews. Syst. Rev..

[57] Kmet L.M., Cook L.S., Lee R.C. (2004). Standard Quality Assessment Criteria for Evaluating Primary Research Papers from a Variety of Fields.

[58] Holmgren H.G., Stockdale L., Gale M., Coyne S.M. (2022). Parent and child problematic media use: The role of maternal postpartum depression and dysfunctional parent-child interactions in young children. Comput. Hum. Behav..

[59] Apisitwasana N., Perngparn U., Cottler L.B. (2018). Effectiveness of school-and family-based interventions to prevent gaming addiction among grades 4–5 students in Bangkok, Thailand. Psychol. Res. Behav. Manag..

[60] Abdullah N.N., Mohamed S., Abu Bakar K., Satari N. (2022). The Influence of Sociodemographic Factors on Mobile Device Use among Young Children in Putrajaya, Malaysia. Children.

[61] Bae E., Choi E.K., Lee H., Kim H. (2020). Factors associated with media addiction in Korean elementary school children. J. Sch. Nurs..

[62] Lim Y., Nam S.J. (2020). Exploring factors related to problematic internet use in childhood and adolescence. Int. J. Ment. Health Addict..

[63] Takahashi M., Adachi M., Nishimura T., Hirota T., Yasuda S., Kuribayashi M., Nakamura K. (2018). Prevalence of pathological and maladaptive Internet use and the association with depression and health-related quality of life in Japanese elementary and junior high school-aged children. Soc. Psychiatry Psychiatr. Epidemiol..

[64] Paulus F.W., Sinzig J., Mayer H., Weber M., von Gontard A. (2018). Computer gaming disorder and ADHD in young children—A population-based study. Int. J. Ment. Health Addict..

[65] De Pasquale C., Chiappedi M., Sciacca F., Martinelli V., Hichy Z. (2021). Online videogames use and anxiety in children during the COVID-19 pandemic. Children.

[66] Aközlü Z., Kolukısa T., Öztürk Şahin Ö., Topan A. (2021). Internet addiction and stressors causing internet addiction in primary school children during the COVID-19 pandemic: A descriptive and cross-sectional study from Turkey. Addicta Turk. J. Addict..

[67] Kroshus E., Tandon P.S., Zhou C., Johnson A.M., Steiner M.K., Christakis D.A. (2022). Problematic Child Media Use During the COVID-19 Pandemic. Pediatrics.

[68] Paulus F.W., Hübler K., Mink F., Möhler E. (2021). Emotional dysregulation in preschool age predicts later media use and Gaming Disorder symptoms in childhood. Front. Psychiatry.

[69] Park J.H., Park M. (2021). Smartphone use patterns and problematic smartphone use among preschool children. PLoS ONE.

[70] Yang H.M., Kim H.R. (2021). Work–Family Conflict on Children’s Internet Addiction: Role of Parenting Styles in Korean Working Mother. Int. J. Environ. Res. Public Health.

[71] Muslu G.K., Aygun O. (2020). An analysis of computer game addiction in primary school children and its affecting factors. J. Addict. Nurs..

[72] Coyne S.M., Shawcroft J., Gale M., Gentile D.A., Etherington J.T., Holmgren H., Stockdale L. (2021). Tantrums, toddlers and technology: Temperament, media emotion regulation, and problematic media use in early childhood. Comput. Hum. Behav..

[73] Eales L., Gillespie S., Alstat R.A., Ferguson G.M., Carlson S.M. (2021). Children’s screen and problematic media use in the United States before and during the covid-19 pandemic. Child Dev..

[74] Korean National Information Society Agency (2011). Third Standardization of Korean Internet Addiction Proneness Scale.

[75] Song H. (2022). Longitudinal Investigations of Autoregressive Cross-Lagged Path Models Among Internet Use, Executive Function Problems, and Maternal Control in Young Korean Children. Front. Psychiatry.

[76] Oh Y., Kim H., Joung Y.S. (2021). Problematic internet use in children according to maternal depression trajectories: A population-based cohort study with 9-year follow-up. J. Psychiatr. Res..

[77] Oh Y., Kim Y., Joung Y.S. (2021). A latent profile analysis of problematic media device use and its association with executive function and behavioral problem among children: A population-based study. Psychiatry Investig..

[78] Horzum M.B., Tuncay A.R.A.S., Balta Ö.Ç. (2008). Computer game addiction scale for children. Turk. Psychol. Couns. Guid. J..

[79] Kök Eren H., Örsal Ö. (2018). Computer game addiction and loneliness in children. Iran. J. Public Health.

[80] Jo S.J., Yim H.W., Lee H.K., Lee H.C., Choi J.S., Baek K.Y. (2018). The Internet Game Use-Elicited Symptom Screen proved to be a valid tool for adolescents aged 10–19 years. Acta Paediatr..

[81] Jeong H., Yim H.W., Lee S.Y., Lee H.K., Potenza M.N., Jo S.J., Son H.J. (2020). A partial mediation effect of father-child attachment and self-esteem between parental marital conflict and subsequent features of internet gaming disorder in children: A 12-month follow-up study. BMC Public Health.

[82] Jeong H., Yim H.W., Lee S.Y., Lee H.K., Potenza M.N., Jo S.J., Son H.J. (2019). Reciprocal relationship between depression and Internet gaming disorder in children: A 12-month follow-up of the iCURE study using cross-lagged path analysis. J. Behav. Addict..

[83] Zhou D., Liu J., Liu J. (2020). The effect of problematic Internet use on mathematics achievement: The mediating role of self-efficacy and the moderating role of teacher-student relationships. Child. Youth Serv. Rev..

[84] Chen S.-H., Weng L.-J., Su Y.-J., Wu H.-M., Yang P.-F. (2003). Development of a Chinese Internet Addiction Scale and Its Psychometric Study. Chin. J. Clin. Psychol..

[85] Hsieh Y.P., Shen A.C.T., Wei H.S., Feng J.Y., Huang S.C.Y., Hwa H.L. (2016). Associations between child maltreatment, PTSD, and internet addiction among Taiwanese students. Comput. Hum. Behav..

[86] Hsieh Y.P., Shen A.C.T., Wei H.S., Feng J.Y., Huang S.C.Y., Hwa H.L. (2018). Internet addiction: A closer look at multidimensional parenting practices and child mental health. Cyberpsychol. Behav. Soc. Netw..

[87] Lo B.C.Y., Lai R.N.M., Ng T.K., Wang H. (2020). Worry and permissive parenting in association with the development of internet addiction in children. Int. J. Environ. Res Public Health.

[88] Miltuze A., Sebre S.B., Martinsone B. (2021). Consistent and Appropriate Parental Restrictions Mitigating Against Children’s Compulsive Internet Use: A One-Year Longitudinal Study. Technol. Knowl. Learn..

[89] Van Petegem S., de Ferrerre E., Soenens B., van Rooij A.J., Van Looy J. (2019). Parents’ degree and style of restrictive mediation of young children’s digital gaming: Associations with parental attitudes and perceived child adjustment. J. Child Fam. Stud..

[90] Yang H., Ng W.Q., Yang Y., Yang S. (2022). Inconsistent Media Mediation and Problematic Smartphone Use in Preschoolers: Maternal Conflict Resolution Styles as Moderators. Children.

[91] Liu S., Yu C., Conner B.T., Wang S., Lai W., Zhang W. (2017). Autistic traits and internet gaming addiction in Chinese children: The mediating effect of emotion regulation and school connectedness. Res. Dev. Disabil..

[92] Richard J., Temcheff C., Fletcher É., Lemieux A., Derevensky J., Déry M. (2022). An empirical investigation of the externalizing and internalizing pathways to disordered gaming behavior: A longitudinal study across childhood and adolescence. Comput. Hum. Behav..

[93] Sakamoto N., Kabaya K., Nakayama M. (2022). Sleep problems, sleep duration, and use of digital devices among primary school students in Japan. BMC Public Health.

[94] Sayı A.K., Şahin F. (2021). Examination of gifted students’ Internet/game addiction and school social behaviors. Pegem J. Educ. Instr..

[95] Kietglaiwansiri T., Chonchaiya W. (2018). Pattern of video game use in children with attention-deficit–hyperactivity disorder and typical development. Pediatr. Int..

[96] Cho K.S., Lee J.M. (2017). Influence of smartphone addiction proneness of young children on problematic behaviors and emotional intelligence: Mediating self-assessment effects of parents using smartphones. Comput. Hum. Behav..

[97] Pamuk M., Atli A. (2016). Development of a Problematic Mobile Phone Use Scale for University Students: Validity and Reliability Study. J. Psychiatry Neurol. Sci..

[98] Pornnoppadol C., Sornpaisarn B., Khamklieng K., Pattana-amorn S. (2014). The Development of Game Addiction Screening Test (GAST). J. Psychiatr. Assoc. Thail..

[99] Shin K.W., Kim D.I., Jung Y.J. (2011). Smartphone Addiction Proneness Scale Development (Research Report).

[100] Yılmaz E., Griffiths M.D., Kan A. (2017). Development and validation of videogame addiction scale for children (VASC). Int. J. Ment. Health Addict..

[101] Mak K.K., Nam J.K., Kim D., Aum N., Choi J.S., Cheng C., Ko H.C., Watanabe H. (2017). Cross-cultural adaptation and psychometric properties of the Korean Scale for Internet Addiction (K-Scale) in Japanese high school students. Psychiatry Res..

[102] Gentile D. (2009). Pathological video-game use among youth ages 8 to 18: A national study. Psychol. Sci..

[103] Meerkerk G.J., Van Den Eijnden R.J., Vermulst A.A., Garretsen H.F. (2009). The Compulsive Internet Use Scale (CIUS): Some psychometric properties. Cyberpsychol. Behav..

[104] Koo H.J., Cho S.H., Kwon J.H. (2015). A study for examining diagnostic ability of the K-Scale as a diagnostic tool for DSM-5 internet gaming disorder. Korean J Clin Psychol..

[105] Kwon S.J., Eum N.R., Korean National Information Society Agency (2016). Digital culture forum policy research report. December 2016. Final Report on Smartphone Overdependence Scale.

[106] Petry N.M., Rehbein F., Gentile D.A., Lemmens J.S., Rumpf H.-J., Mößle T., Bischof G., Tao R., Fung D.S.S., Borges G. (2014). An international consensus for assessing internet gaming disorder using the new DSM-5 approach. Addiction.

[107] Ayas T., Çakır Ö., Horzum M.B. (2011). Adolescent’s Computer Addiction Scale. Kast. Educ. J..

[108] Van Rooij A.J., Schoenmakers T.M., van den Eijnden R.J., van de Mheen D. (2012). Videogame Addiction Test (VAT): Validity and psychometric characteristics. Cyberpsychol. Behav. Soc. Netw..

[109] Kwon M., Kim D.J., Cho H., Yang S. (2013). The smartphone addiction scale: Development and validation of a short version for adolescents. PLoS ONE.

[110] Dahl D., Bergmark K.H. (2020). Persistence in Problematic Internet Use—A Systematic Review and Meta-Analysis. Front. Sociol..

[111] Lee C., Lee S.-J. (2017). Prevalence and predictors of smartphone addiction proneness among Korean adolescents. Child. Youth Serv. Rev..

[112] Dong C., Cao S., Li H. (2020). Young children’s online learning during COVID-19 pandemic: Chinese parents’ beliefs and attitudes. Child. Youth Serv. Rev..

[113] Burriss K.G., Tsao L.L. (2002). Review of research: How much do we know about the importance of play in child development?. Child. Educ..

[114] Bochicchio V., Maldonato N.M., Valerio P., Vitelli R., dell’Orco S., Scandurra C. A review on the effects of digital play on children’s cognitive and socio-emotional development. Proceedings of the 2018 9th IEEE International Conference on Cognitive Infocommunications (CogInfoCom).

[115] World Health Organization (2019). Guidelines on Physical Activity, Sedentary Behaviour and Sleep for Children under 5 Years of Age.

[116] Hill D., Ameenuddin N., Chassiakos Y.R., Cross C., Hutchinson J., Levine A., Boyd R., Mendelson R., Moreno M., AAP Council on Communations and Media (2016). Media and Young Minds. Pediatrics.

[117] Donati M.A., Sanson F., Mazzarese M., Primi C. (2019). Assessing video game habits and pathological behaviour in children through a new scale: Psychometric properties of the Video-Gaming Scale—For Children (VGS-C). Psychology.

[118] Hawi N.S., Samaha M., Griffiths M.D. (2019). The digital addiction scale for children: Development and validation. Cyberpsychol. Behav. Soc. Netw..

[119] Barker V. (2009). Older adolescents’ motivations for social network site use: The influence of gender, group identity, and collective self-esteem. Cyberpsychol. Behav..

[120] Kuss D.J., Griffiths M.D. (2011). Online social networking and addiction—A review of the psychological literature. Int. J. Environ. Res. Public Health.

[121] Durkee T., Kaess M., Carli V., Parzer P., Wasserman C., Floderus B., Apter A., Balazs J., Barzilay S., Bobes J. (2012). Prevalence of pathological internet use among adolescents in E urope: Demographic and social factors. Addiction.

[122] Gentile D.A. (2011). The multiple dimensions of video game effects. Child Dev. Perspect..

[123] Desai R.A., Krishnan-Sarin S., Cavallo D., Potenza M.N. (2010). Video-gaming among high school students: Health correlates, gender differences, and problematic gaming. Pediatrics.

[124] Leonhardt M., Overå S. (2021). Are there differences in video gaming and use of social media among boys and girls?—A mixed methods approach. Int. J. Environ. Res. Public Health.

[125] Li W., Garland E.L., Howard M.O. (2014). Family factors in Internet addiction among Chinese youth: A review of English-and Chinese-language studies. Comput. Hum. Behav..

[126] D’Arienzo M.C., Boursier V., Griffiths M.D. (2019). Addiction to social media and attachment styles: A systematic literature review. Int. J. Ment. Health Addict..

[127] Bussone S., Trentini C., Tambelli R., Carola V. (2020). Early-life interpersonal and affective risk factors for pathological gaming. Front. Psychiatry.

[128] Ghinassi S., Casale S. (2023). The Role of Attachment in Gambling Behaviors and Gambling Disorder: A Systematic Review. J. Gambl. Stud..

[129] Schimmenti A., Caretti V. (2010). Psychic retreats or psychic pits? Unbearable states of mind and technological addiction. Psychoanal. Psychol..

[130] Schimmenti A., Guglielmucci F., Barbasio C.P., Granieri A. (2012). Attachment disorganization and dissociation in virtual worlds: A study on problematic Internet use among players of online role playing games. Clin. Neuropsychiatry.

[131] Schimmenti A., Passanisi A., Gervasi A.M., Manzella S., Famà F.I. (2014). Insecure attachment attitudes in the onset of problematic Internet use among late adolescents. Child Psychiatry Hum. Dev..

[132] Estevez A., Jauregui P., Lopez-Gonzalez H. (2019). Attachment and behavioral addictions in adolescents: The mediating and moderating role of coping strategies. Scand. J. Psychol..

[133] Throuvala M.A., Janikian M., Griffiths M.D., Rennoldson M., Kuss D.J. (2019). The role of family and personality traits in Internet gaming disorder: A mediation model combining cognitive and attachment perspectives. J. Behav. Addict..

[134] Sung Y., Nam T.H., Hwang M.H. (2020). Attachment style, stressful events, and Internet gaming addiction in Korean university students. Pers. Individ. Differ..

[135] Gioia F., Rega V., Boursier V. (2021). Problematic Internet use and emotional dysregulation among young people: A literature review. Clin. Neuropsychiatry.

[136] Bowlby J. (1973). Attachment and Loss: Vol. 2. Separation: Anxiety and Anger.

[137] Ainsworth M.D.S., Blehar M.C., Waters E., Wall S. (1978). The Strange Situation: Observing Patterns of Attachment.

[138] Kardefelt-Winther D. (2014). A conceptual and methodological critique of internet addiction research: Towards a model of compensatory internet use. Comput. Hum. Behav..

[139] Bowlby J. (1982). Attachment and loss: Retrospect and prospect. Am. J. Orthopsychiatry.

[140] Thompson R.A. (1994). Emotion regulation: A theme in search of definition. Monogr. Soc. Res. Child Dev..

[141] Cassidy J. (1994). Emotion regulation: Influences of attachment relationships. Monogr. Soc. Res. Child Dev..

[142] Fonagy P., Gergely G., Jurist E.L., Target M. (2002). Affect Regulation, Mentalization, and the Development of the Self.

[143] Schultheis A.M., Mayes L.C., Rutherford H.J. (2019). Associations between emotion regulation and parental reflective functioning. J. Child Fam. Stud..

[144] Gordon-Hacker A., Gueron-Sela N. (2020). Maternal use of media to regulate child distress: A double-edged sword? Longitudinal links to toddlers’ negative emotionality. Cyberpsychol. Behav. Soc. Netw..

[145] Sasson H., Mesch G.S. (2019). Parental mediation. Int. Encycl. Media Lit..

[146] Nielsen P., Favez N., Liddle H., Rigter H. (2019). Linking parental mediation practices to adolescents’ problematic online screen use: A systematic literature review. J. Behav. Addict..

[147] Rega V., Gioia F., Boursier V. (2022). Parental Mediation and Cyberbullying: A Narrative Literature Review. Marriage Fam. Rev..

[148] Raffaelli M., Crockett L.J., Shen Y.L. (2005). Developmental stability and change in self-regulation from childhood to adolescence. J. Genet. Psychol..

[149] Blos P. (1967). The second individuation process of adolescence. Psychoanal. Study Child.

[150] Lukavská K., Hrabec O., Lukavský J., Demetrovics Z., Király O. (2022). The associations of adolescent problematic internet use with parenting: A meta-analysis. Addict. Behav..

[151] Fam J.Y., Männikkö N., Juhari R., Kääriäinen M. (2022). Is parental mediation negatively associated with problematic media use among children and adolescents? A systematic review and meta-analysis. Can. J. Behav. Sci..

[152] Liau A.K., Neo E.C., Gentile D.A., Choo H., Sim T., Li D., Khoo A. (2015). Impulsivity, self-regulation, and pathological video gaming among youth: Testing a mediation model. Asia Pac. J. Public Health.

[153] Billieux J., Van der Linden M. (2012). Problematic use of the Internet and self-regulation: A review of the initial studies. Open Addict. J..

[154] Grolnick W.S., Farkas M., Bornstein M.H. (2002). Parenting and the development of children’s self-regulation. Handbook of Parenting: Practical Issues in Parenting.

[155] Dwairy M., Achoui M. (2010). Parental control: A second cross-cultural research on parenting and psychological adjustment of children. J. Child Fam. Stud..

