# Supplementary material for: Problematic Media Use among Children up to the Age of 10: A Systematic Literature Review
Source: Int J Environ Res Public Health. 2023 May 17;20(10):5854. doi: 10.3390/ijerph20105854 (PMC10217802; doi:10.3390/ijerph20105854)
Supplement: Supplementary file 1 [file ijerph-20-05854-s001.zip › ijerph-2283912-supplementary.pdf]

Supplementary Materials

Problematic Media Use among Children up to the Age of 10: A Systematic Literature Review

Table S1. *Quality assessment of the reviewed studies using the QUALSYST evaluation tool (Author 1).*

STUDIES	Q1	Q2	Q3	Q4	Q5	Q6	Q7	Q8	Q9	Q10	Q11	Q12	Q13	Q14	SUMMARY SCORE
ABDULLAH ET AL. (2022)	1	2	0	2	n/a	n/a	n/a	1	2	2	2	1	2	2	17/22=0.77
AKÖZLÜ ET AL. (2021)	1	2	1	1	n/a	n/a	n/a	2	1	2	2	n/a	1	2	15/20=0.75
APISITWASANA ET AL. (2018)	1	2	2	1	n/a	n/a	n/a	1	1	2	2	1	2	2	17/22=0.77
BAE ET AL. (2020)	1	2	2	2	n/a	n/a	n/a	2	2	2	2	1	2	2	20/22=0.91
CHO ET AL. (2017)	2	0	1	2	n/a	n/a	n/a	1	1	2	2	1	2	1	15/22=0.68
COYNE ET AL. (2021)	2	2	2	2	n/a	n/a	n/a	2	2	2	2	2	2	2	22/22=1.00
DE PASQUALE ET AL. (2021)	2	2	1	2	n/a	n/a	n/a	2	1	1	2	0	2	2	17/22=0.77
EALES ET AL. (2021)	2	1	2	2	n/a	n/a	n/a	2	2	2	2	1	2	2	20/22=0.91
HOLMGREN ET AL. (2022)	2	2	2	2	n/a	n/a	n/a	2	2	2	1	2	2	2	21/22=0.95
HSIEH ET AL. (2016)	2	1	2	1	n/a	n/a	n/a	2	2	1	1	1	2	2	17/22=0.77
HSIEH ET AL. (2018)	2	1	2	1	n/a	n/a	n/a	2	2	2	2	2	2	2	20/22=0.91
JEONG ET AL. (2019)	1	1	1	2	n/a	n/a	n/a	2	2	2	2	2	2	2	19/22=0.86
JEONG ET AL. (2020)	2	1	2	2	n/a	n/a	n/a	2	1	2	2	2	2	2	20/22=0.91
KIETGLAIWANSIRI ET AL. (2018)	1	1	2	2	n/a	n/a	n/a	1	1	2	1	2	2	2	17/22=0.77
KÖK EREN & ÖRSAL (2018)	1	1	0	2	n/a	n/a	n/a	1	1	2	1	n/a	1	2	12/20=0.60
KROSHUS ET AL. (2022)	2	1	2	2	n/a	n/a	n/a	2	2	2	2	1	2	2	20/22=0.91
LIM ET AL. (2020)	2	1	2	2	n/a	n/a	n/a	0	2	2	2	1	2	2	18/22=0.82

**LIU ET AL. (2017)**	2	2	2	2	n/a	n/a	n/a	2	2	2	2	2	2	2	22/22=1.00
**LO ET AL. (2020)**	2	2	2	1	n/a	n/a	n/a	2	2	2	2	0	2	2	19/22=0.86
**MILTUZE ET AL. (2021)**	2	2	1	2	n/a	n/a	n/a	1	2	2	1	1	2	2	18/22=0.82
**MUSLU ET AL. (2020)**	1	2	1	2	n/a	n/a	n/a	2	2	2	2	n/a	2	2	18/20=0.90
**OH ET AL. (2021A)**	1	2	2	1	n/a	n/a	n/a	1	2	2	2	1	2	2	18/22=0.82
**OH ET AL. (2021B)**	1	2	2	1	n/a	n/a	n/a	2	2	2	2	1	1	2	18/22=0.82
**PARK ET AL. (2021)**	1	2	2	2	n/a	n/a	n/a	2	2	2	2	1	2	2	20/22=0.91
**PAULUS ET AL. (2018)**	1	2	1	2	n/a	n/a	n/a	1	2	2	2	1	2	2	18/22=0.82
**PAULUS ET AL. (2021)**	2	2	1	2	n/a	n/a	n/a	2	1	2	2	0	2	2	18/22=0.82
**RICHARD ET AL. (2022)**	2	2	2	2	n/a	n/a	n/a	1	2	2	2	2	2	2	21/22=0.95
**SAKAMOTO ET AL. (2022)**	1	2	2	2	n/a	n/a	n/a	1	2	2	2	1	2	2	19/22=0.86
**SAYI ET AL. (2021)**	2	2	2	2	n/a	n/a	n/a	1	1	2	2	n/a	2	2	18/20=0.90
**SONG (2022)**	1	2	2	2	n/a	n/a	n/a	2	2	2	2	1	2	2	20/22=0.91
**TAKAHASHI ET AL. (2018)**	2	2	2	2	n/a	n/a	n/a	2	2	2	2	1	2	2	21/22=0.95
**VAN PETEGEM ET AL. (2019)**	2	2	2	2	n/a	n/a	n/a	2	2	2	2	2	2	2	22/22=1.00
**YANG ET AL. (2021)**	2	2	2	2	n/a	n/a	n/a	2	2	2	2	2	2	2	22/22=1.00
**YANG ET AL. (2022)**	2	1	0	2	n/a	n/a	n/a	2	1	2	2	2	2	2	18/22=0.82
**ZHOU ET AL. (2020)**	2	2	2	2	n/a	n/a	n/a	2	2	2	2	2	2	2	22/22=1.00

2 = yes; 1 = partial; 0 = no; n/a = not applicable.

Table S2. *Quality assessment of the reviewed studies using the QUALSYST evaluation tool (Author 2).*

STUDIES	Q1	Q2	Q3	Q4	Q5	Q6	Q7	Q8	Q9	Q10	Q11	Q12	Q13	Q14	SUMMARY SCORE
**ABDULLAH ET AL. (2022)**	1	2	1	1	n/a	n/a	n/a	1	2	1	2	1	2	2	16/22=0.73
**AKÖZLÜ ET AL. (2021)**	1	2	1	1	n/a	n/a	n/a	2	1	1	2	n/a	1	2	14/20=0.70
**APISITWASANA ET AL. (2018)**	1	2	2	1	n/a	n/a	n/a	1	2	2	2	1	2	2	18/22=0.81
**BAE ET AL. (2020)**	1	2	2	1	n/a	n/a	n/a	2	2	2	2	1	2	2	19/22=0.86
**CHO ET AL. (2017)**	2	0	1	2	n/a	n/a	n/a	1	1	2	2	1	2	1	15/22=0.68
**COYNE ET AL. (2021)**	2	2	2	2	n/a	n/a	n/a	2	2	2	2	2	2	2	22/22=1.00
**DE PASQUALE ET AL. (2021)**	1	1	1	1	n/a	n/a	n/a	2	1	1	2	0	2	2	14/22=0.63
**EALES ET AL. (2021)**	2	2	2	2	n/a	n/a	n/a	2	2	2	2	1	2	2	21/22=0.95
**HOLMGREN ET AL. (2022)**	2	2	2	2	n/a	n/a	n/a	2	2	2	2	2	2	2	22/22=1.00
**HSIEH ET AL. (2016)**	2	1	1	1	n/a	n/a	n/a	2	2	1	1	1	2	2	16/22=0.73
**HSIEH ET AL. (2018)**	2	1	2	1	n/a	n/a	n/a	2	2	2	2	2	2	2	20/22=0.91
**JEONG ET AL. (2019)**	1	1	1	2	n/a	n/a	n/a	2	2	2	2	2	2	2	19/22=0.86
**JEONG ET AL. (2020)**	1	1	2	2	n/a	n/a	n/a	2	2	2	2	2	2	2	20/22=0.91
**KIETGLAIWANSIRI ET AL. (2018)**	1	2	2	2	n/a	n/a	n/a	1	2	1	2	2	2	2	19/22=0.86
**KÖK EREN & ÖRSAL (2018)**	1	1	0	2	n/a	n/a	n/a	1	1	1	1	n/a	1	2	11/20=0.55
**KROSHUS ET AL. (2022)**	2	1	1	2	n/a	n/a	n/a	2	2	2	1	2	2	2	19/22=0.86
**LIM ET AL. (2020)**	2	1	2	1	n/a	n/a	n/a	1	2	1	1	1	1	2	15/22=0.68
**LIU ET AL. (2017)**	2	2	2	1	n/a	n/a	n/a	2	2	2	2	2	2	2	21/22=0.95
**LO ET AL. (2020)**	2	2	1	1	n/a	n/a	n/a	2	2	2	2	0	2	2	18/22=0.82

**MILTUZE ET AL. (2021)**	2	2	1	2	n/a	n/a	n/a	1	2	2	2	1	2	2	19/22=0.86
**MUSLU ET AL. (2020)**	1	2	1	2	n/a	n/a	n/a	2	2	2	2	n/a	2	2	18/20=0.90
**OH ET AL. (2021A)**	1	2	2	1	n/a	n/a	n/a	1	2	2	2	1	2	2	18/22=0.82
**OH ET AL. (2021B)**	1	2	2	1	n/a	n/a	n/a	2	2	2	2	1	1	2	18/22=0.82
**PARK ET AL. (2021)**	1	1	2	2	n/a	n/a	n/a	2	2	2	2	1	2	2	19/22=0.86
**PAULUS ET AL. (2018)**	1	1	1	2	n/a	n/a	n/a	1	2	2	2	1	2	2	17/22=0.77
**PAULUS ET AL. (2021)**	2	2	1	2	n/a	n/a	n/a	2	2	2	2	0	2	2	19/22=0.86
**RICHARD ET AL. (2022)**	2	2	2	2	n/a	n/a	n/a	1	2	2	2	2	2	2	21/22=0.95
**SAKAMOTO ET AL. (2022)**	1	2	2	2	n/a	n/a	n/a	1	2	2	2	1	2	2	19/22=0.86
**SAYI ET AL. (2021)**	2	2	2	2	n/a	n/a	n/a	1	1	1	2	n/a	2	2	17/20=0.85
**SONG (2022)**	2	2	2	2	n/a	n/a	n/a	2	2	2	2	1	2	2	21/22=0.95
**TAKAHASHI ET AL. (2018)**	2	2	2	2	n/a	n/a	n/a	2	2	2	2	1	2	2	21/22=0.95
**VAN PETEGEM ET AL. (2019)**	2	1	1	2	n/a	n/a	n/a	2	2	2	2	2	2	2	20/22=0.91
**YANG ET AL. (2021)**	1	1	1	2	n/a	n/a	n/a	2	2	2	2	2	2	2	19/22=0.86
**YANG ET AL. (2022)**	2	1	0	2	n/a	n/a	n/a	2	1	2	2	2	2	2	18/22=0.82
**ZHOU ET AL. (2020)**	2	2	2	2	n/a	n/a	n/a	2	2	2	2	2	2	2	22/22=1.00

2 = yes; 1 = partial; 0 = no; n/a = not applicable.

Table S3. *Inter-rater agreement and disagreement for overall scores of quantitative studies.*

STUDIES	SUMMARY SCORE (R1)	SUMMARY SCORE (R2)	DISCREPANCIES
**ABDULLAH ET AL. (2022)**	17/22=0.77	16/22=0.73	0.04
**AKÖZLÜ ET AL. (2021)**	15/20=0.75	14/20=0.70	0.05
**APISITWASANA ET AL. (2018)**	17/22=0.77	18/22=0.81	0.04
**BAE ET AL. (2020)**	20/22=0.91	19/22=0.86	0.05
**CHO ET AL. (2017)**	15/22=0.68	15/22=0.68	0
**COYNE ET AL. (2021)**	22/22=1.00	22/22=1.00	0
**DE PASQUALE ET AL. (2021)**	17/22=0.77	14/22=0.63	0.14
**EALES ET AL. (2021)**	20/22=0.91	21/22=0.95	0.04
**HOLMGREN ET AL. (2022)**	21/22=0.95	22/22=1.00	0.05
**HSIEH ET AL. (2016)**	17/22=0.77	16/22=0.73	0.04
**HSIEH ET AL. (2018)**	20/22=0.91	20/22=0.91	0
**JEONG ET AL. (2019)**	19/22=0.86	19/22=0.86	0
**JEONG ET AL. (2020)**	20/22=0.91	20/22=0.91	0
**KIETGLAIWANSIRI ET AL. (2018)**	17/22=0.77	19/22=0.86	0.09
**KÖK EREN & ÖRSAL (2018)**	12/20=0.60	11/20=0.55	0.05
**KROSHUS ET AL. (2022)**	20/22=0.91	19/22=0.86	0.05
**LIM ET AL. (2020)**	18/22=0.82	15/22=0.68	0.14
**LIU ET AL. (2017)**	22/22=1.00	21/22=0.95	0.05

**LO ET AL. (2020)**	19/22=0.86	18/22=0.82	0.04
**MILTUZE ET AL. (2021)**	18/22=0.82	19/22=0.86	0.04
**MUSLU ET AL. (2020)**	18/20=0.90	18/20=0.90	0
**OH ET AL. (2021A)**	18/22=0.82	18/22=0.82	0
**OH ET AL. (2021B)**	18/22=0.82	18/22=0.82	0
**PARK ET AL. (2021)**	20/22=0.91	19/22=0.86	0.05
**PAULUS ET AL. (2018)**	18/22=0.82	17/22=0.77	0.05
**PAULUS ET AL. (2021)**	18/22=0.82	19/22=0.86	0.04
**RICHARD ET AL. (2022)**	21/22=0.95	21/22=0.95	0
**SAKAMOTO ET AL. (2022)**	19/22=0.86	19/22=0.86	0
**SAYI ET AL. (2021)**	18/20=0.90	17/20=0.85	0.05
**SONG ET AL. (2022)**	20/22=0.91	21/22=0.95	0.04
**TAKAHASHI ET AL. (2018)**	21/22=0.95	21/22=0.95	0
**VAN PETEGEM ET AL. (2019)**	22/22=1.00	20/22=0.91	0.09
**YANG ET AL. (2021)**	22/22=1.00	19/22=0.86	0.14
**YANG ET AL. (2022)**	18/22=0.82	18/22=0.82	0
**ZHOU ET AL. (2020)**	22/22=1.00	22/22=1.00	0

R1, Reviewer 1; R2, Reviewer 2.
